# Weekends admitted adult medical patients have higher in-hospital mortality in Ethiopia: An implication for quality improvement

**DOI:** 10.1371/journal.pone.0312538

**Published:** 2024-10-24

**Authors:** Balew Arega, Gashaw Solela, Elias Tewabe, Asnake Agunie, Amanuel Zeleke, Ermiyas Tefera, Abraham Minda, Yitagesu Getachew

**Affiliations:** 1 Department of Internal Medicine, Yekatit 12 Hospital Medical College, Addis Ababa, Ethiopia; 2 Department Health Care Quality, Yekatit 12 Hospital Medical College, Addis Ababa, Ethiopia; 3 Meri Prime Clinic, Addis Ababa, Ethiopia; Imperial College London School of Public Health, UNITED KINGDOM OF GREAT BRITAIN AND NORTHERN IRELAND

## Abstract

**Background:**

Weekend effect’ is a term used to describe the increased mortality associated with weekend emergency admissions to hospitals compared with admissions on weekdays. This effect was not investigated in Ethiopia among adult patients admitted to hospitals. We aimed to find out whether the weekend effect exists in the country’s a teaching hospital.

**Methods:**

The study was conducted among adult medical patients admitted at Yekatit 12 Hospital Medical College, Addis Ababa, Ethiopia, from September 2020 to September 2023. We extracted the data from the electronic medical records, and those with missed outcomes, length of hospital stays, and diagnosis were excluded. We used a multivariable logistic regression model to determine the association between the outcome and risk factors. The Cox proportional hazard model was utilized to establish the correlation between admission times and mortality risk incidence. Statistical significance was determined using a P value of less than 0.05. The Kaplan-Meier curve was utilized to estimate the risk of in-hospital mortality over the duration of the hospital stay.

**Results:**

A of 5564 patients were admitted to medical wards (n = 5001) and intensive care unit (ICU) (n = 563) during the study periods. In binary multivariable analysis, weekend medical wards and ICU admitted adult medical patients had a 38% (AOR, 1.38, 95% CI, 1.17, 1.65) and 50% (AOR, 1.50, 95% CI, 1.02, 2.20) higher in-hospital mortality compared weekdays admitted patients, respectively. The cumulative mortality risk incidence was higher among medical ward-admitted patients (AHR, 1.26, 95% CI, 0.09, 1.46, P value = 0.051) and significantly higher among ICU-admitted adult patients (AHR, 1.28, 95% CI, 1.21, 1.75, P value = 0.01) during the weekends. There was no statistically significant mortality difference among night versus day or office hours versus off-office hours admitted patients. Moreover, we did identify significant differences in the duration of hospital stays between weekends and weekdays.

**Conclusions:**

In this study, weekend-admitted adult medical patients in wards or ICUs have higher in-hospital mortality rates. This underscores a need for comprehensive nationwide data to improve weekend admitted patients’ quality of care and treatment outcomes.

## Background

The weekend effect, the phenomenon of patients admitted at the weekend having a higher mortality risk, has been widely investigated and documented [1]. Since the 1970s, researchers have found worse outcomes for patients hospitalized or treated on weekends [2–4]. In addition, recent observational studies [5,6] and meta-analyses [7,8] reported a weekend effect. However, studies undertaken on specific types of patients, such as those with heart failure and stroke, showed no mortality difference between individuals hospitalized on the weekends [9,10]. Much debate surrounds the suspected reasons behind the weekend effect, including patient-specific variables such as the severity of illness, number of comorbidity factors, or patient extrinsic factors including reduced weekend staffing and increased delays in time to necessary interventions [11–13] or due to a lack of supervision or inconsistency in the quality of care [14].

The weekend effect was extensively studied in high-income countries, and the majority of them reported higher mortality among weekend-admitted patients [15–17]. However, few studies conducted in developing countries reported different findings [18,19]. For example, a study in Kenya found that hospital patients treated on weekends had similar death rates to those admitted on weekdays [19]. This similarity may reflect a stable level of care or a generalized shortage of resources and staffing that subsumes any impact of weekly variations. Another study in Tanzania [18], however, showed a significantly higher mortality risk for patients admitted during the weekend. Given that healthcare systems worldwide are heterogeneous [20], local evidence is of paramount importance in investigating the weekend effect on patient outcomes.

The weekend effect could be a significant concern in Ethiopia [15]. In northern Ethiopia, for instance, the study found that pediatric patients admitted on weekends had a 63% higher death rate 15]. However, with an extensive search using PubMed, Embase, Scopus, Google Scholar, Web of Science, Midline, and others, we did not fund a study investigating the weekend effect among adult patients in the country of Ethiopia. Therefore, we sought to study the weekend effect on patients’ mortality and length of hospital stays by analyzing three years of adult patient data from electronic medical records (EMR) at a teaching hospital in Addis Ababa, Ethiopia. The findings have room for quality improvement projects designed to address the weekend effect on patient outcomes.

## Methods

### Study area, design, and period

Ethiopia has a three-tiered health system, including primary, secondary, and tertiary care. The number of health professionals, healthcare providers’ expertise profile, available investigation, and level of care provided differed based on the healthcare level [23]. Across all levels, off-office patient care is provided by duty-time healthcare personnel. During duty time, the number of healthcare providers providing care is reduced by at least half, despite considerable variation depending on the institution’s nature. Healthcare workers cover the duty hours alternatively [21].

We performed a retrospective study using the medical records of all adult patients admitted at Yekatit 12 Hospital Medical College (Y12HMC), Ethiopia, from September 2020 to September 2023. It is one of the largest teaching hospitals in the country, with over 500 beds, treating approximately 310, 000 patients each year, and having more than 21 departments. The internal medicine department comprises six medical wards and an adult intensive care unit (ICU). Patients are admitted to the inpatient units of the hospital either through referring institutions or the hospital’s emergency or regular outpatient departments. The hospital started electronic medical records (EMR) in late 2019 and is currently implementing it across inpatient care, outpatient care, laboratory, imaging studies, and pharmacy services. Every patient’s medical record is documented in the EMR, and authorized staff can access it. The EMR has required fields such as gender, age, and recording of the date and time of services rendered. The database of patient records with specified information can be obtained in Excel format. We extracted data from adult medical patients who were admitted to the wards and ICU of the hospital during the study period.

### Data source, variables, and outcome

We retrieved retrospective data from the EMR system and accessed it from January 23 to 25, 2024. The variables included in this study were age, gender, time of admission, days of hospitalization, admission disease categorization, length of hospital stay (LOS), and in-hospital mortality. Using the International Classification of Diseases and Clinical Modification (ICD-10-CM), we classified the admission diagnosis into systems [22]. The study excluded patients’ data for whom essential variables (outcome, diagnosis, date of discharge, date of admission, sex, and age) were missed in their medical records. In the data collection, the specific patients identified (name and medical record number (MRN)) were not accessed and collected. The admission time was categorized as a weekend (Saturday and Sunday), weekdays (Monday to Friday), office hours, off-office hours, early night, late night, nighttime, and daytime. Off-office hours include the weekday nighttime, holidays, and weekends. Inpatient mortality is when the patient dies from admission to discharge. The duration from admission to discharge is the LOS. The operational definition is presented in S1 Table. The outcome was in-hospital mortality among hospitalized patients in ICUs and wards, stratified by admission time or days.

### Ethical considerations

Since we used the retrospective data archived in the EMR, there is no need for consent to be obtained. Any identifier of the specific patients was not included in the publication. The Yekatit 12 Hospital Medical College Research Ethical Committee waived ethical approval; however, the College provided a formal letter permitting the data to be used for publication and further analysis.

### Statistical analysis

The data collected from the electronic medical record in Excel format was imported into SPSS version 27, where we cleansed the data before the data analysis proceeded. We performed the analysis using STATA, version 16.1 (StataCorp, Texas, USA). Baseline characteristics were summarized using descriptive statistics. The outlier was checked using the data normalization after trimming 5%, which was almost equivalent. The LOS was summarized in median and interquartile ranges. The sociodemographic and clinical characteristics of patients admitted during the weekend and weekdays were compared using an independent sample proportion. We used binary multivariable logistics regression to determine the association between outcome variables and risk factors. The Cox proportional hazard model (LOS is a time variable) was used to determine the effect of time of admission on patient mortality. All variables with a P value ≤ 0.2 in the crude analysis of these regressions will be entered into the multivariate regressions, and a P value <0.05 was used to declare statistical significance. The Kaplan-Meier curve was used to estimate the in-hospital mortality over the length of the hospital stay.

## Results

### Description of hospital admissions

**Fig 1:**
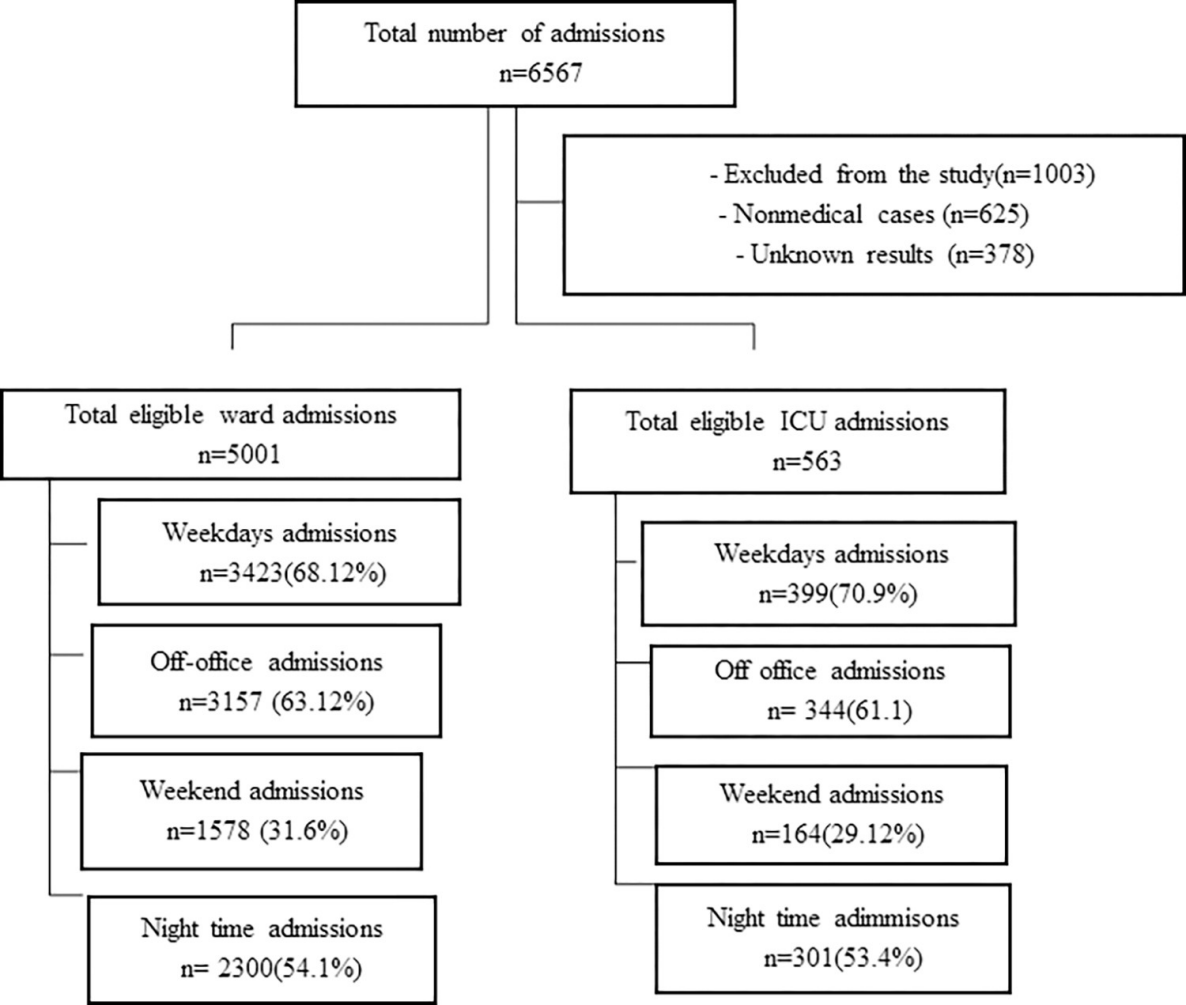
Flow chart of the study populations.

From September 2020 and September 2023, a total of 6567 (5,942 medical and 625 non-medical cases) adult patients were admitted to the medical wards and ICU. We excluded 625(9.2%) non-medical cases (surgical patients) admitted to medical wards because of the bed-sharing rule of the hospital. Among 5,942 medical cases, 378 (6.4%) were excluded because of missed documentation of LOS and outcomes and missed diagnoses. In the final analysis, we consisted of 5564 patients (response rate: 93.6%), 5001 patients from the wards, and 563 from the ICU. Of ward-admitted patients, 3157 (63.1%) were admitted during off-office hours and 2300 (54.1%) during the night period. Among ICU-admitted patients, 344 (61.1%) were hospitalized during off-office hours and 301 (53.4%) at night (Fig 1).

### Baseline characteristics of adult patients admitted to medical wards

In Medical wards, 31.6% (1578/5564) and 72.45% (3423/5564) patients were admitted during weekends and weekdays respectively. Slightly more than half (51.1%) were female, and the mean age was 49.5 years. Circulatory system, infectious, and respiratory diseases were the three top ICD-10 disease classification causes of admission, accounting for 31.9%(1595/5001), 29.3% (1464/5001), and 9.60% (480/5001), respectively (Table 1). There were statistically significant differences between patients with ICD-10 disease classification (P = 0.001) but not based on age and gender concerning the time of admission (weekends vs weekdays) (Table 1).

**Table 1:** Disease classification and sociodemographic characteristics of patients admitted to the wards in a teaching hospital in Ethiopia.

Variables	Number of ward admissions				Mortality rate		
	Total	Weekends n (%)	Weekdays n (%)	P value	Weekends n (%)	Weekdays n (%)	P value
**Sex**				0.83			0.42
Male	2426(48.5)	769(48.9)	1567(48.4)		128(52.7)	212(53.4)	
Female	2575(51.5)	809(51.8)	1766 (51.6)		115(47.3)	185(46.6)	
**Age groups**				0.92			0.79
<25	479(9.6)	152(9.6)	327(9.6)		22(9.1)	33(8.3)	
25–45	1785(35.7)	566(35.9)	1219(35.6)		94(38.7)	143(36)	
46–65	1507(30.1)	491(31.1)	1016(29.6)		71(29.2)	114(28.7)	
>65	1230(24.6)	369(23.4)	861(25.2)		56(23)	107(27)	
**Disease classification (ICD-10)**				0.6			0.001
Circulatory system	1595(31.9)	505(32.1)	1090(31.9)		74(30.5)	136(34.3)	
Infectious diseases	1464(29.3)	462(29.3)	1002(29.3)		93(38.3)	157(39.5)	
Respiratory system	480(9.60)	128(8.1)	352(9.6)		31(12.8)	30(7.6)	
Endocrine and metabolic diseases	396(7.90)	164(10.4)	232(7.9)		8(3.3)	7(1.8)	
Blood and blood-forming organs	379(7.60)	139(8.8)	240(7.6)		10(4.4)	16(4.0)	
Digestive system	357(7.10)	82(5.2)	275(7.1)		17(7.0)	28(7.1)	
Genitourinary system	215(4.30)	59(3.7)	156(4.3)		7(2.9)	17(4.3)	
Nervous system	82(1.60)	31(2.0)	51(1.6)		2(2.9)	4(1.0)	
**Musculoskeletal system**	33(1.2)	8(0.5)	25(0.7)		1(0.4)	2(0.5)	

### Baseline characteristics of adult medical patients admitted to ICU

Among ICU-admitted medical patients, 70.9% (399/563) were admitted on weekdays, and 29.1% (164/563) were admitted on weekends. About half (50.44%) were female, and the mean age was 50.13 years. Cardiovascular disease was the major cause of ICU admissions, accounting for 35.70% (201/563), followed by infectious diseases at 21.7% (121/563) and respiratory disorders at 10.80% (61/563). Based on the time of admission, patients were significantly different based on ICD-disease classification(p = 0.001) but not with sex or age (Table 2).

**Table 2:** Sociodemographic characteristics and disease classification of patients admitted to the ICU in a teaching hospital in Ethiopia.

Variables	Number of ward admissions				Mortality rate		
	Total	Weekends n (%)	Weekdays n (%)	P Value	Weekends, n (%)	Weekdays n (%)	P value
** Sex**				0.11			0.145
Male	279(49.55)	90 (54.9)	189(47.4)		38(61.3)	56(48.7)	
Female	284(50.44)	74(45.1)	210(52.8)		24(38.7)	59(53.3)	
**Age groups**				0.96			0.54
<25	55(9.8)	17(10.4)	38(9.6)		8(12.8)	9(7.8)	
25–45	192(34.1)	60(36.6)	132(33.1)		22(35.5)	44(38.3)	
46–65	168(29.8)	48(29.3)	120(30.1)		18(19.0)	32(27.8)	
>65	148(26.3)	39(23.8)	109(26.3)		14(22.6)	30(26.1)	
**Disease classification (ICD-10)**				0.78			0.001
Circulatory system	201(35.7)	58(35.2)	201(35.8)		19(30.6)	28(24.4)	
Infectious diseases	121(21.7)	29(17.6)	121(21.5)		20(32.3)	46(40.0)	
Respiratory system	61(10.80)	13(7.9)	61(10.9)		1(1.6)	10(10.8)	
Endocrine and metabolic diseases	53(9.40)	26(15.8)	53(9.4)		2(3.2)	2(1.7)	
Blood and blood-forming organs	42(7.50)	13(7.9)	42(7.5)		8(8.5)	8(7.0)	
Digestive system	45(8.00)	16(9.7)	45(8.0)		11(17.7)	14(12.2)	
Genitourinary system	30(5.30)	6(3.6)	30(5.3)		2(3.2)	4(3.5)	
Nervous system	7(1.20)	3(1.8)	7(1.2)		1(1.6)	2(1.7)	
Musculoskeletal System	3(0.62)	1(0.6)	2(0.4)		1(1.6)	1(0.9)	

### Mortality of adult medical patients admitted to medical wards

**Fig 2:**
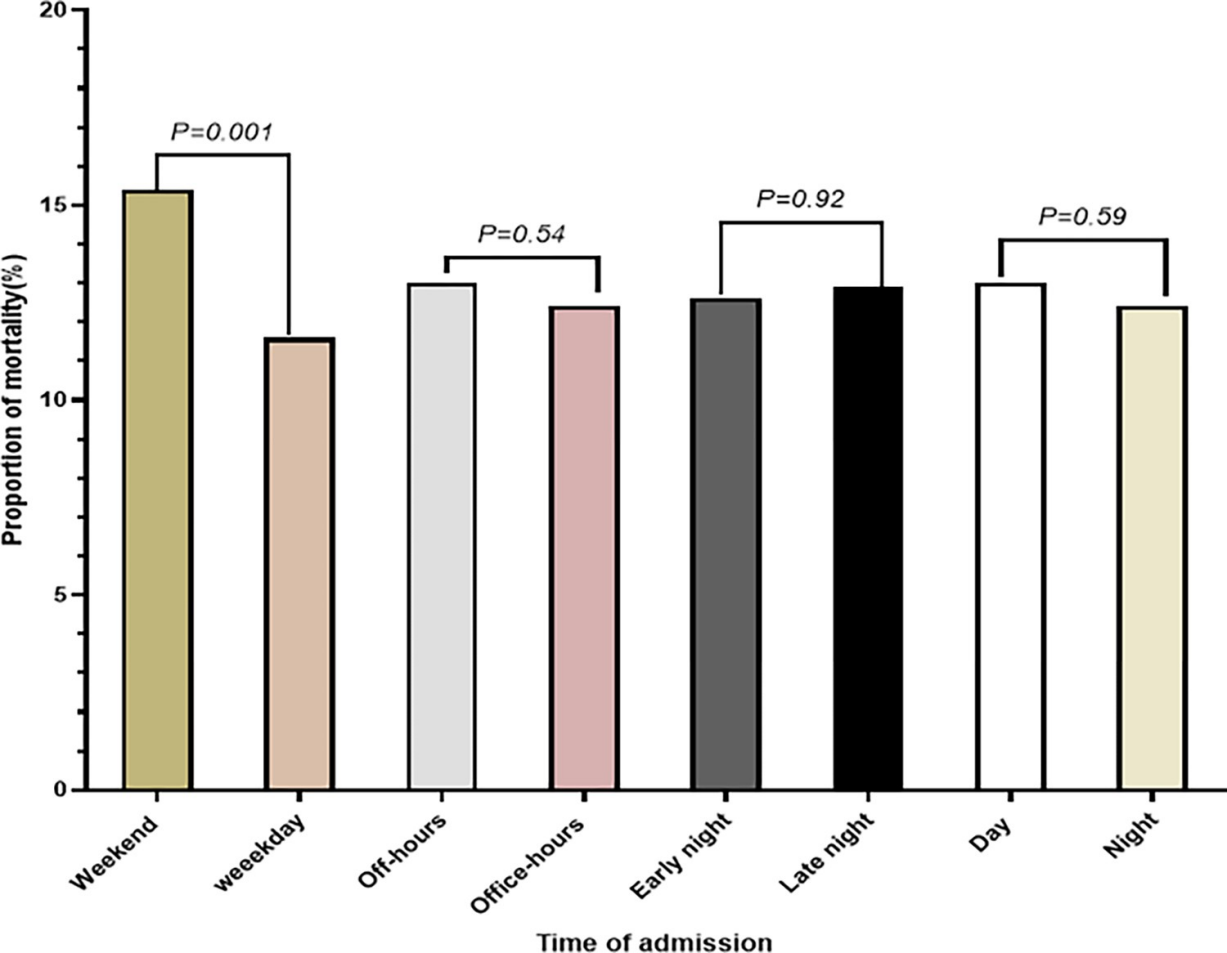
The mortality rate and its association with the time of admissions among adult patients admitted to medical wards in a teaching hospital in Ethiopia.

The overall in-hospital mortality rate of adult patients admitted to medical wards was 12.8% (640/5564). In the binary multivariable regression, adult patients admitted to medical patients during the weekend had 38% higher in-hospital odds of dying as compared with patients admitted on the weekdays, and this difference was found to be statistically significant (AOR, 1.38, 95% CI, 1.17, 1.65) (S2 Table and Fig 2). However, there was no statistically significant difference in mortality rate between patients admitted on daytime vs nighttime admissions, office vs off-office hour admissions, and early night vs late nighttime weekend and weekday admissions (S2 Table and Fig 2). The details findings of univariate and multivariate binary multivariable logistic regression are present in S2 Table.

**Fig 3:**
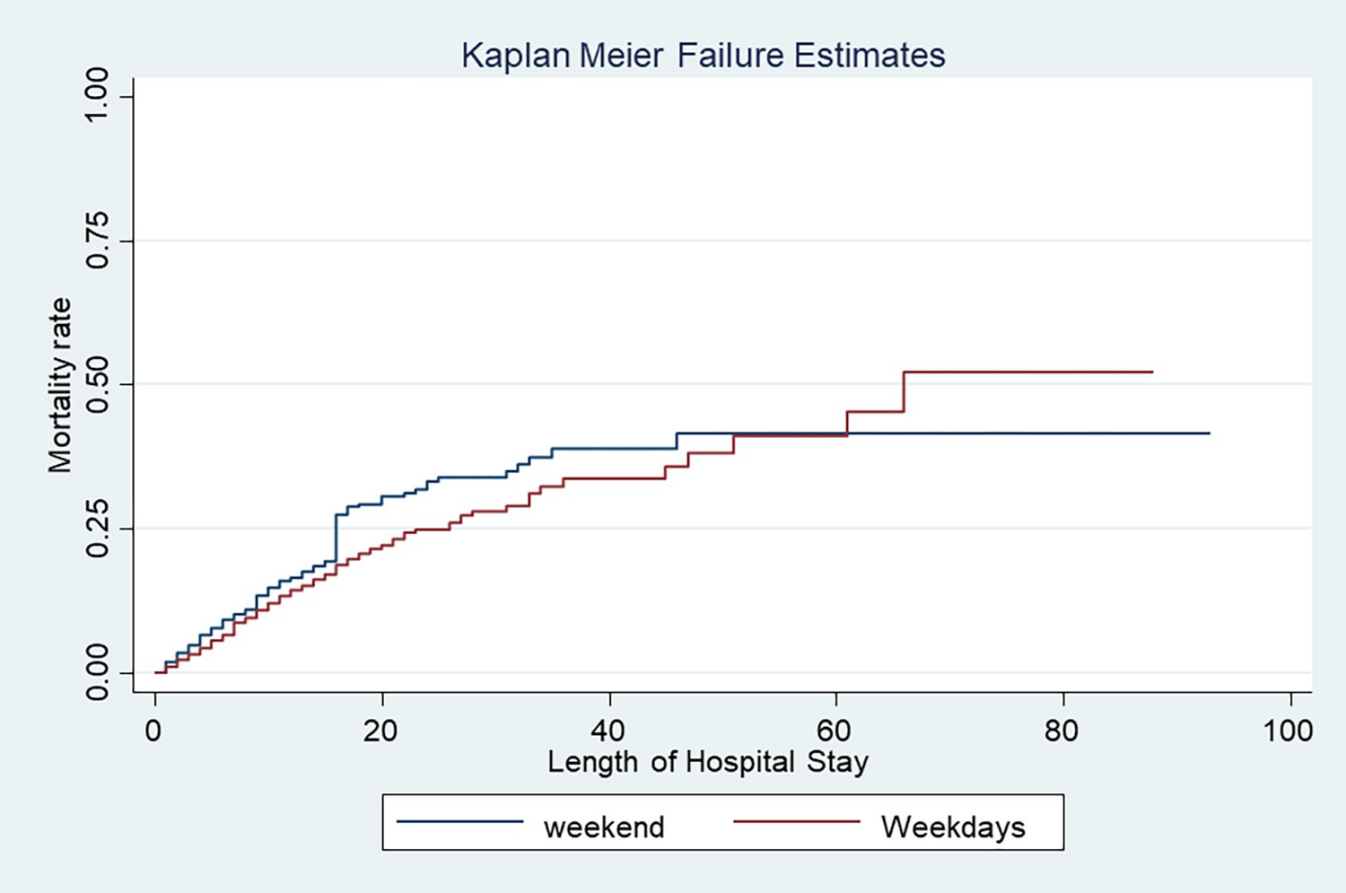
Kaplan-Meier curves showing cumulative mortality risk against the length of hospital stay among medical wards adult patients in a teaching hospital, in Ethiopia.

The log-rank tests indicate that mortality risk was higher among patients admitted to the wards during the weekend as compared to the weekday-admitted patients (log-rank, 2.88, P value, 0.09) (Fig 3). The multiple Cox proportional hazards model showed an increased cumulative risk of death among medical wards on weekends admitted adult patients compared with those admitted on weekdays (HR = 1.26, 95% CI, 0.09, 1.46, P value = 0.051). We present the details of the Cox proportional hazard model analysis in S2 Table for patients admitted to the medical ward.

### Morality of adult medical patients admitted to the ICU

**Fig 4:**
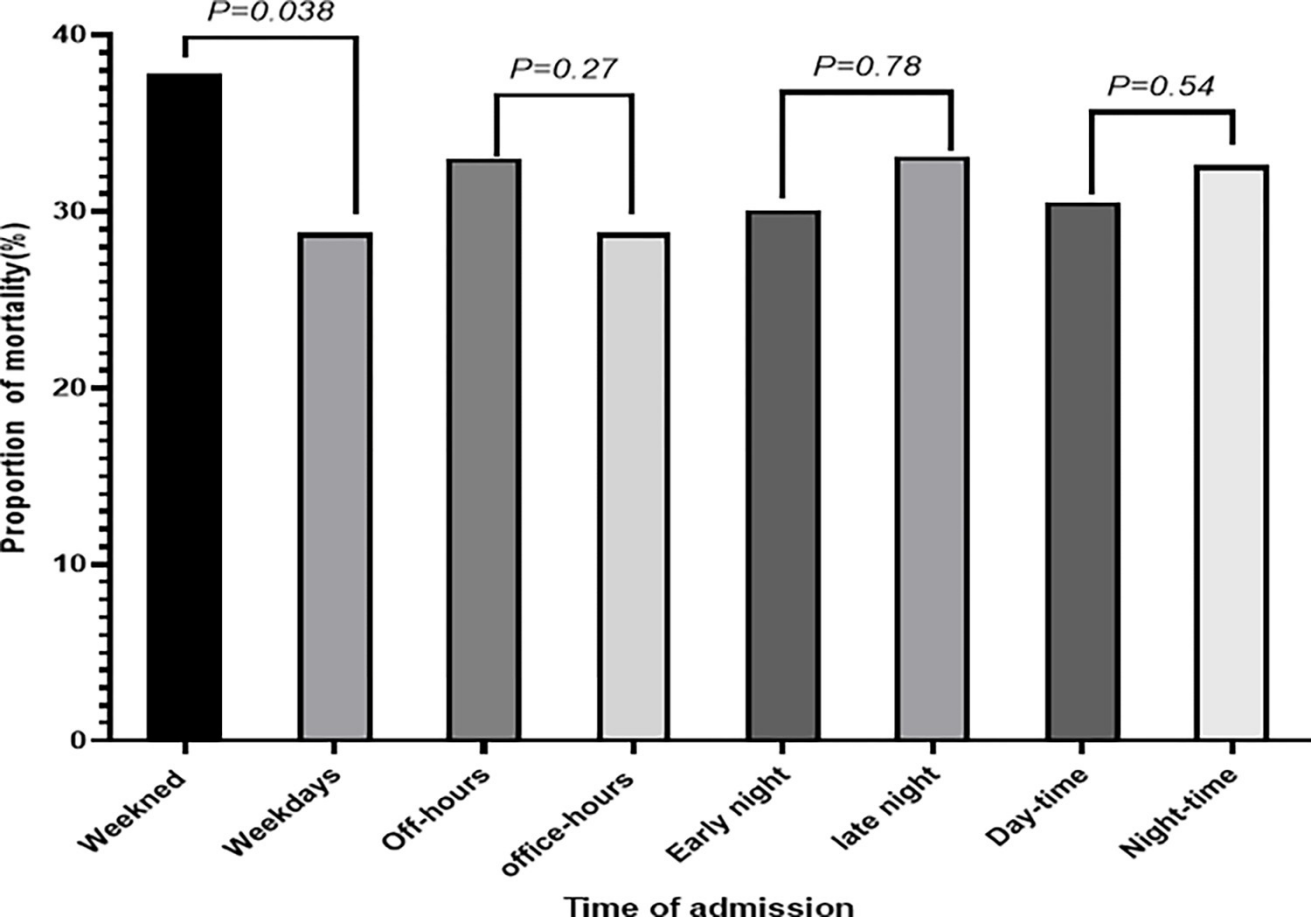
Mortality rate and its association with the time of admissions among adult medical patients admitted in ICU in a teaching hospital in Ethiopia.

In the ICU admissions, the overall in-hospital mortality rate was 31.4% (177/563). According to the binary logistic regression analysis, adult weekend admitted medical patients in-hospital odds of dying were 50% higher than those of patients admitted on weekdays. This was statistically significant (AOR, 1.50, 95% CI, 1.02, 2.20) (S3 Table and Fig 4). However, in-hospital odds of dying were not significantly different between daytime vs nighttime, office hours vs off-office hours, or early vs late night admissions. The details of ICU-admitted adult medical patients’ univariate and multivariate binary logistic regression findings are present in S3 Table.

**Fig 5:**
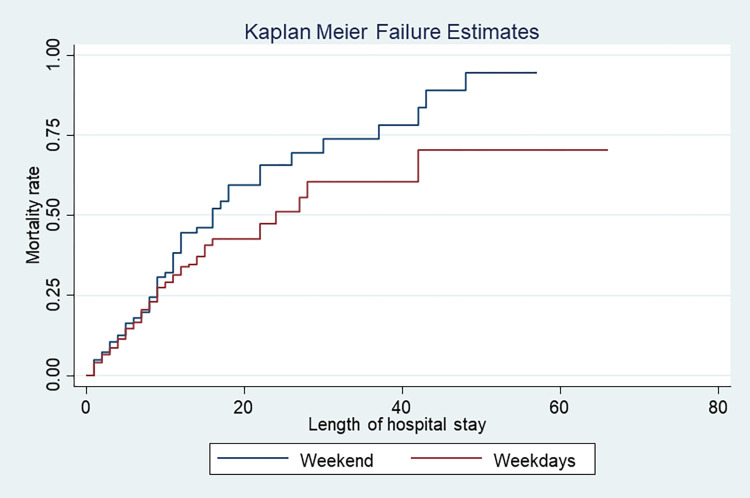
Kaplan-Meier curves showing cumulative mortality risk against LOS among adult medical patients admitted to the ICU in teaching hospitals in Ethiopia.

The log-rank tests reveal that the mortality risk was greater among ICU weekend-admitted adult medical patients as compared to the weekday-admitted adult patients (Log-Rank, 9.52, P value, 0.02). In the multiple Cox proportional hazard model, patients admitted during the weekend had a significantly higher cumulative risk of death compared to those admitted on the weekdays (AHR, 1.28, 95% CI, 1.21, 1.75, P value = 0.01). Fig 5 illustrates the cumulative mortality risk of the weekend and weekday ICU-admitted medical patients using the Kaplan-Meier analysis and log-rank test. The binary and Cox proportional hazard model analysis of adult medical patients admitted to the ICU is depicted in S3 Table.

### Length of hospital stay

The overall (wards and ICU) median LOS for adult medical patients admitted on the weekends (7 days) was not significantly different from those admitted on the weekdays (8 days) (P value = 0.06). The median LOS of adult patients admitted to medical wards was not significantly different (P value = 0.06) between the weekends (7 days) and weekdays (8 days). However, we cannot determine the median LOS difference of adult medical patients admitted to ICU during weekends vs weekdays. This is because the patients initially admitted to the ICU were transferred to the wards, and we could not trace their stay in the ICU from the EMR system.

## Discussion

We used the EMR system data in a teaching hospital to assess the weekend effect on adult medical patients’ in-hospital mortality and their length of stay in the medical wards and ICU. We found significantly higher in-hospital mortality among adult patients admitted during the weekends to the wards and ICU. However, the LOS was not significantly different between the times of the admissions.

We found that patients admitted to medical wards on weekends had a significantly higher in-hospital mortality risk than those admitted on weekdays. This is comparable to the findings of two meta-analyses, which included a large number of individual studies (ranging from 39 to 97) from high-income countries [23,24]. Both meta-analyses revealed that patients admitted on the weekends had significantly higher overall mortality rates than weekday-admitted patients. In our study, the greater death risk during weekend admission might be attributed to the following conditions: First, the number of healthcare providers during the weekend is reduced by at least half, which might impede the effective handling of emergency conditions and result in higher mortality [23]. In a previous study, reduced staffing was found to be a major driver of the excess mortality risk associated with weekend admissions [23]. In another study, however, there was no significant difference in mortality for weekend patients when staffing was similar to that of weekdays [24]. Second, working on the weekend is unpopular and lacks a consistent level of morality for patient care. In other studies, health professionals were less satisfied with weekend time and duty-hour-based patient care, which might affect their productivity and patients’ outcomes [25,26]. Third, patients hospitalized on weekends may be sicker, leading to greater in-hospital mortality. However, studies conducted elsewhere found that the weekend effect was higher after adjusting for the severity of the illness [17]. The association between weekend admissions and higher mortality rates has important implications for healthcare resource allocation and staffing decisions in low-resource settings, including in our setting. In addition, synthesizing our findings with other studies on healthcare quality in Ethiopia [30,31], we suggest that addressing the weekend effect is crucial for improving overall healthcare outcomes in the country.

In our study, the in-hospital mortality risk of adult medical patients admitted to the ICU on weekends was significantly higher than that of weekday admissions. Previously conducted studies had mixed findings that were non-significant in older studies [27,28], but significantly higher in-hospital mortality was reported in a recent systematic review and meta-analysis [29]. The heterogenicity of the weekend effect on ICU patient mortality across the studies might be related to the facility’s ICU infrastructure, staffing, location, and teaching status of hospitals, the severity of disease, and the presence of coexisting illnesses among admitted patients. Our findings suggest that the weekend effect among patients admitted to the general wards and ICU highlights the need for targeted interventions to address the disparity. In another study [30], the availability of intensivists (pulmonary critical care specialists) has been found to reduce the mortality rate of a patient in the ICU setting. However, these specialists are not available in our setting, and ICU care has been given by internists, emergency physicians, and anesthesiologists. Designing quality improvement projects addressing organizational factors [31], specific quality projects such as easy action principles [32], duty hours telemedicine [33], and others were found to significantly reduce ICU patient mortality during off-office hours. Implementing projects in other countries may not be feasible in our setting as the majority of factors are institutional-based. However, this study might serve as a baseline for the weekend impact seen in Ethiopia, allowing us to identify specific factors for quality enhancement programs.

Similar to the findings of the present study, other studies found no significant difference in the death rates whether the patients were admitted at daytime or nighttime and on-office or off-office hours to the ICU [34]. However, some studies showed a higher mortality rate for patients admitted during the nighttime [30,35]. Consistent with another study [36], we did not find a significant difference in the LOS based on the time of admission. The absence of a significant difference in the mortality rates of patients admitted at night versus daytime might be acceptable. In our hospital, we have observed that patients were hospitalized mainly in the late daytime and early nighttime (up to 6:00 AM); the admitted cases were presented in the upcoming morning sessions, and they got the chance for better evaluation by the full treating team in the office hours in the immediate morning. However, those patients admitted on the weekends stayed for a minimum of 2.5 days (about 60 hours) before they were evaluated by the full treatment team. This might have contributed to the presence of higher in-hospital mortality in those patients admitted on the weekends.

There are some limitations to our study. First, patient information was collected from retrospective administrative patient data, which might have had incomplete and missing data. The majority of the patients excluded from the study were non-medical patients’ data who were admitted to the medical ward according to the hospital’s bed-sharing principle. Since we included only adult medical cases, these patients are not outside population groups. However, about six percent of medical patients had missing data and were therefore excluded from the study. This study has relatively low data incompleteness since we gathered a small number of variables, the majority of which are also necessary fields in the EMR. In addition, we grouped the diagnoses based on the ICD-10 classification by extracting the main diagnosis from the EMR initially submitted by the treating physician during the patient’s hospitalization. In individuals with several comorbidities, the potential for misclassification increases. The second limitation is that we were unable to identify whether the outcomes were related to quality of care or weekend staffing, nor could we provide explanations for the reasons for the weekend effect. Since we used administrative data and important potential confounders were missed, we could only draw very cautious conclusions concerning the root causes behind the observations.

## Conclusions

This study, the first of its kind in Ethiopia to assess the weekend effect, demonstrated that patients admitted to the wards or ICU on the weekends had a higher in-hospital mortality as compared with patients admitted on the weekdays. A prospective study investigating the role of patient characteristics, staffing effect, and healthcare processes in different hospital settings would provide a deeper understanding of the weekend effect in our country’s context. The findings of such investigations ultimately improve the quality of care and treatment outcomes for hospitalized patients in our country.

## Supporting information

S1 Table Operational definitions. (DOCX)

S2 Table Binary and Cox proportional regressions analysis in-hospital mortality in association to time of admission of adult patients admitted to medical ward-admitted. Legends: Vs = versus, COR = Crude odd ratio, AOR = Adjusted odd ratio, CHR = Crude hazard ratio, AHR = adjusted Hazard ratio, CI = Confidence interval, *all have p-value less 0.2 in COR and CHR, ^+^statistically significant.
(DOCX)

S3 Table Binary and Cox proportional regressions analysis in-hospital mortality in association to time of admission of adult patients admitted to intensive care unit. Legends: Vs = Versus COR = Crude odd ratio, AOR = Adjusted odd ratio, CHR = Crude hazard ratio, AH = adjusted Hazard ratio, CI = Confidence interval *all have p value less 0.2 in COR and CHR, ^+^statistically significant.
(DOCX)

## Abbreviations

AHR: Adjusted Harvard ration
AOR: Adjusted Odd ration
CKD: Chronic kidney disease
CLD: Chronic liver disease
HIV: Human immune deficiency virus
LOS: Length of Hospital stay
NCD: Noncommunicable disease
