# Supplementary material for: Weekends admitted adult medical patients have higher in-hospital mortality in Ethiopia: An implication for quality improvement
Source: PLoS One. 2024 Oct 24;19(10):e0312538. doi: 10.1371/journal.pone.0312538 (PMC11500864; doi:10.1371/journal.pone.0312538)
Supplement: S1 Table — (DOCX) [file pone.0312538.s001.docx]

| Time | Definition |
| --- | --- |
| Weekday | Monday (from 8:30 AM), to Friday (up to 8:30 AM); |
| Weekend | Saturday (8:30 AM) to Sunday (8:30 AM) |
| Office hour | Working hours during the working days or weekdays (not including the holidays) |
| Off-office hours | Nighttime (5:30 PM to 8:30 AM) of weekdays, both day and night time of weekends and holidays. |
| Nighttime | The time from 5:30 PM to 8:30 AM |
| Daytime | The time from 8:30 AM to 5:30 PM |
| Length of hospital stay: | The period between the patient's admission and discharge |
| Early night | The time from 5:00 PM to 12:00 AM |
| Late night | The time from 12:00 AM to 6:00 AM |
| In-hospital mortality | Refers to the in-hospital death of a patient after admission to the wards or ICU and it is taken as an event. |
| Censured | Refers to a patient who went against medical advice, discharged cured or improved, referred or left |
