# Supplementary material for: Weekends admitted adult medical patients have higher in-hospital mortality in Ethiopia: An implication for quality improvement
Source: PLoS One. 2024 Oct 24;19(10):e0312538. doi: 10.1371/journal.pone.0312538 (PMC11500864; doi:10.1371/journal.pone.0312538)
Supplement: S3 Table — Legends: Vs = Versus COR = Crude odd ratio, AOR = Adjusted odd ratio, CHR = Crude hazard ratio, AH = adjusted Hazard ratio, CI = Confidence interval *all have p value less 0.2 in COR and CHR, +statistically significant. (DOCX) [file pone.0312538.s003.docx]

| **Variables** | Binary logistic regression of ward mortality | | | Cox proportional hazard model of ward mortality | | |
| --- | --- | --- | --- | --- | --- | --- |
|  | COR (95%CI) | AOR (95%CI) | P value | CHR (95% CI) | AHR (95%CI) | P value |
| **Weekdays vs weekend admissions** |  |  |  |  |  |  |
| Weekdays | 1 | 1 |  |  |  |  |
| Weekend | 1.34(1.02,1.45)* | 1.50 (1.17,1.65) | 0.038 | 1.24 (1.17,1.65) * | 1.28 (1.19, 1.46) | 0.01^+^ |
| **Office vs. off-office hour admissions** |  |  |  |  |  |  |
| Office-hour | 1 | 1 |  | 1 | 1 |  |
| Off-office | 1.05(0.91, 1.25) * | 1.17(0.23, 1.44) | 0.54 | 1.11(0.91, 1.34) * | 1.11(0.81, 1.44) * | 0.49 |
| **Days vs. night admissions** |  |  |  |  |  |  |
| Daytime | 1 | 1 |  | 1 | 1 |  |
| Night time | 1.24(0.82,1.55) * | 1.22(0.52,1.75) | 0.59 | 1.40(0.52,1.47) * | 1.06(0.62,1.77) * | 0.36 |
| **Early vs. late-night admissions** |  | 1 |  | 1 | 1 |  |
| Early night | 1 |  |  |  |  |  |
| Late night | 1.21(0.59,1.63) * | 1.17(0.39,1.54) | 0.92 | 1.34(0.42,1.51) * | 1.13(0.22,1.55) * | 0.44 |
